# Supplementary material for: Atmospheric CO2 effect on stable carbon isotope composition of terrestrial fossil archives
Source: Nat Commun. 2018 Jan 17;9:252. doi: 10.1038/s41467-017-02691-x (PMC5772509; doi:10.1038/s41467-017-02691-x)
Supplement: Supplementary file 1 — Supplementary Information [file 41467_2017_2691_MOESM1_ESM.pdf]

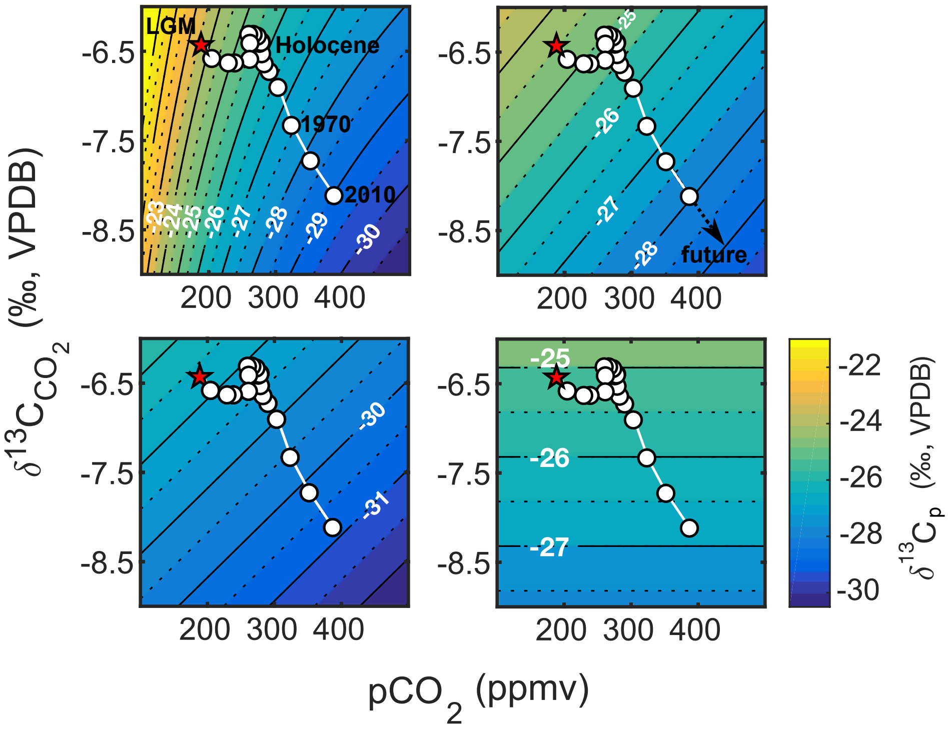

Supplementary Figure 1. Effects of pCO_2 and $\delta^{13}C_{CO_2}$ on the stable carbon isotope composition of C_3 land plants according to four different models. Identical white curves indicate the trajectory of $\delta^{13}C_p$ from the Last Glacial Maximum (LGM, indicated by red stars) until 2010 C.E., which is implied by ice core records and historical atmospheric measurements. Note that white dots represent 20 year intervals from 1950 onwards. Before 1950 they represent 1000 year intervals. Black arrows indicate predicted direction of $\delta^{13}C_p$ under future climate change. Top left, hyperbolic model SJ-2012 [1]. Top right, Voelker-2016g [2]. Bottom left, Voelker-2016a. Bottom right, version of the Farquhar-1982 model [3], assuming constant c_i/c_a by stomatal regulation of leaf gas-exchange. Additional historical data is taken from [4], which covers the most recent period up to 2010 C.E.

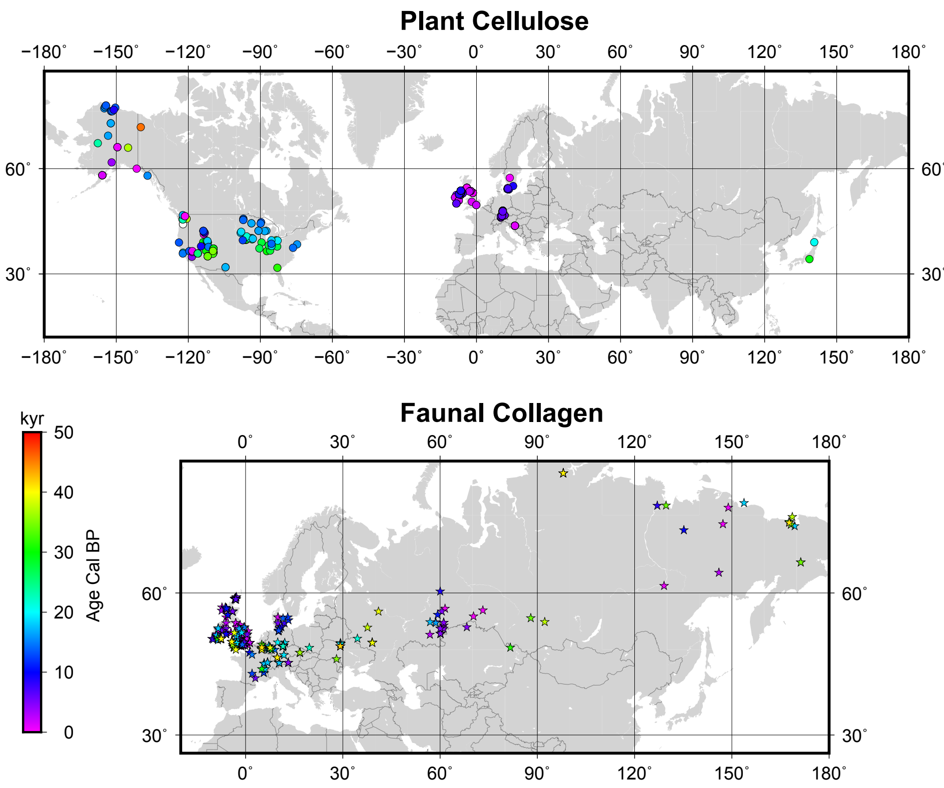

Supplementary Figure 2. Geographical distribution of plant and faunal δ^{13}C records. Filled circles represent plant cellulose, and filled stars show fauna from predominantly C_3 ecosystems in western Europe and northern Eurasia. The colour indicates the radiocarbon date in kyr cal BP (BP = 1950 CE), inset is the colour bar with age scale. Note that for plant cellulose, there are relatively few records represented from western Europe before 15 kyr, when most of the data are from North America and Japan. Faunal collagen, on the other hand, show reasonably good temporal coverage over a wide geographic range.

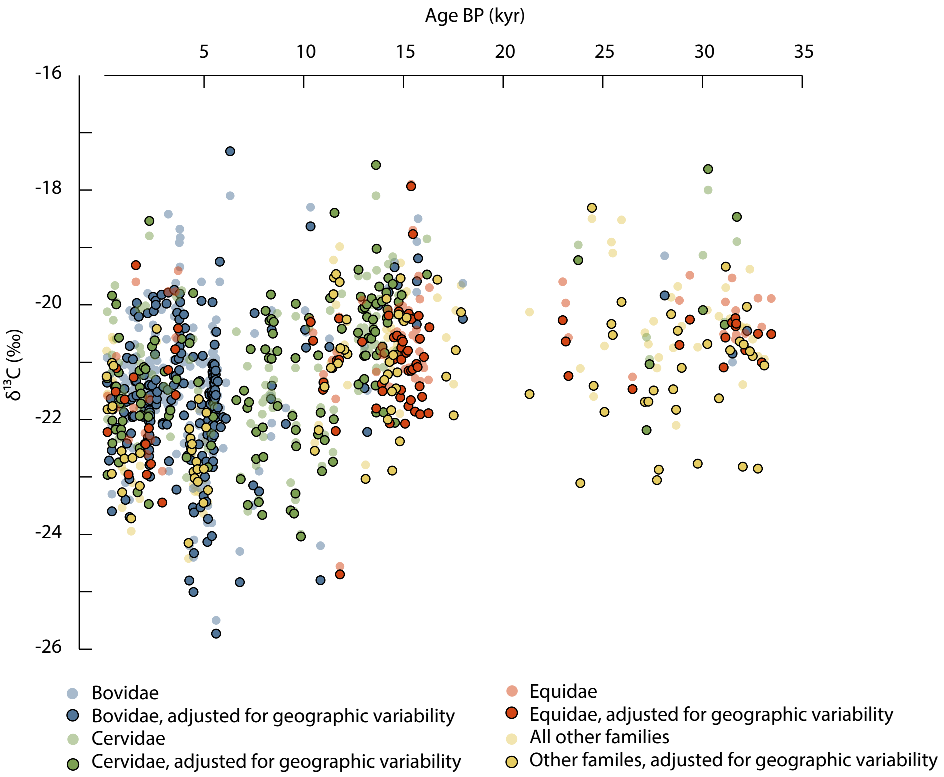

Supplementary Figure 3. Faunal δ^{13}C records plotted by group along with adjustments for MAP/altitude/latitude. Ungulates are reasonably well represented across the past 35 kyr, with the exception of Bovidae, for which records are denser before 6 kyr. Other faunal species (yellow circles) are slightly more negative than the large ungulates across the record, which is most likely due to a slightly smaller collagen-diet enrichment factor, $\varepsilon^*_{\text{d-c}}$, for small mammals. We do not attempt to correct for $\varepsilon^*_{\text{d-c}}$ for two reasons. First, the difference is small, and accurate values of $\varepsilon^*_{\text{d-c}}$ are difficult to obtain for a variety of small mammals with different physiologies. Second, both ungulates and small mammals are fairly evenly represented across the dataset, which means that there is a negligible effect on the calculation of the shift in mean δ^{13}C calculated between $<$ 20 kyr and $<$ 10 kyr.

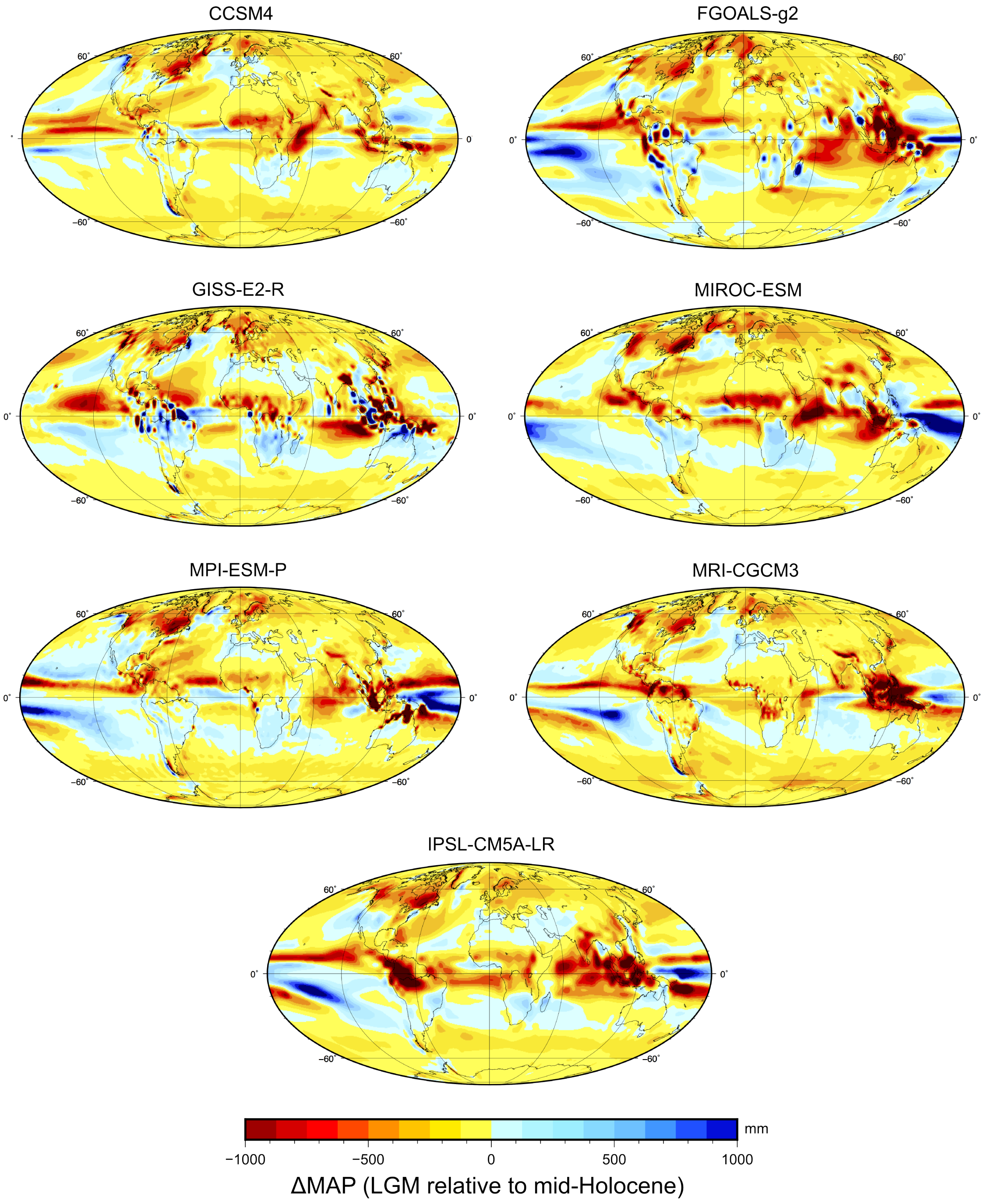

Supplementary Figure 4. PMIP3-CMIP5 models of MAP change between the Mid-Holocene (6 kyr) and the LGM (21 kyr) included in this study. Data were obtained for the following seven models (available from https://pmip3.lsce.ipsl.fr, accessed June 2016): CCSM4, University of Miami, U.S.A.; FGOALS-g2, LASG, Institute of Atmospheric Physics, Chinese Academy of Sciences and CESS, Tsinghua University, China ; GISS-E2-R, NASA Goddard Institute for Space Studies, U.S.A.; IPSL-CM5A-LR, Institut Pierre-Simon Laplace, France; MIROC-ESM, Japan Agency for Marine-Earth Science and Technology, Atmosphere and Ocean, Japan Research Institute (The University of Tokyo), and National Institute for Environmental Studies, Japan; MPI-ESM-P, Max-Planck-Institut für Meteorologie (Max Planck Institute for Meteorology), Germany; MRI-CGCM3, Meteorological Research Institute, Japan.)

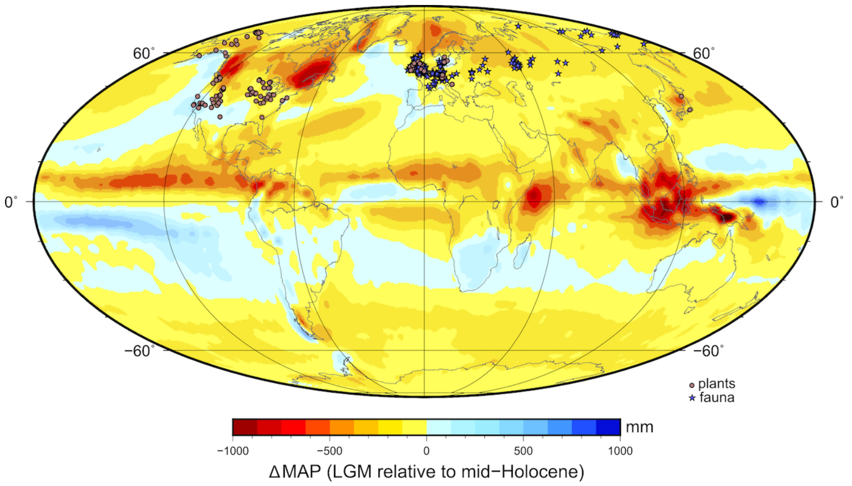

Supplementary Figure 5. PMIP3-CMIP5 ensemble model of MAP change between the Mid-Holocene (6 kyr) and the LGM (21 kyr). All plants are represented by brown circles. Fauna are blue stars.

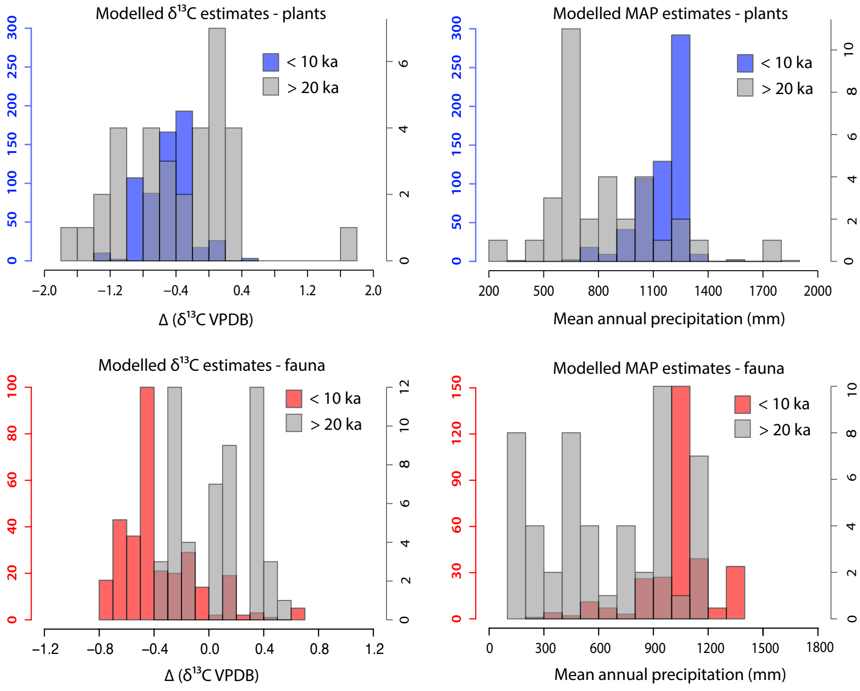

Supplementary Figure 6. Histograms of MAP change and corresponding shift in δ^{13}C interpolated for our dataset using PMIP3-CMIP5 model ensemble. The magnitude of predicted change in δ^{13}C ranges from +0.67 to −0.78 ‰ (fauna) and +0.13 to −1.72 ‰ (gymnosperms).

Supplementary Table 1. Statistics for comparisons of photosynthetic models of fractionation with all adjusted plant cellulose data. $\Delta(\delta^{13}C)$ indicates the magnitude of the difference in $\delta^{13}C_p$ between 20 kyr and 10 kyr which is predicted by each model.

Model	$\Delta(\delta^{13}C)$, ‰	RMSE	AIC	BIC
SJ-2012	–1.97	1.038	304	318
Volker-2016a	–0.66	2.965	2438	2456
Volker-2016g	–0.79	1.153	375	394
Farquhar-1982	–0.10	1.249	437	451

Supplementary Table 2. Statistics for comparisons of photosynthetic models of fractionation with gymnosperm plant cellulose data only.

Model	RMSE	AIC	BIC
SJ-2012	1.073	25	31
Volker-2016a	3.242	185	193
Volker-2016g	1.044	26	34
Farquhar-1982	1.170	29	34

Supplementary Table 3. Statistics for model comparisons with equivalent plant $\delta^{13}C$ derived from adjusted faunal $\delta^{13}C$ with the subtraction of ε^*_{d-c} = 5.1 ‰.

Model	RMSE	AIC	BIC
SJ-2012	1.718	598	611
Volker-2016a	2.058	857	874
Volker-2016g	1.819	671	688
Farquhar-1982	1.836	682	695

Supplementary Table 4. Average contributions of changing MAP, $\Delta(\delta^{13}\bar{C}_{MAP})$, and pCO_2, $\Delta(\delta^{13}\bar{C}_{pCO_2})$, to the total shift in stable carbon isotope records between > 20 kyr and < 10 kyr (excluding the industrial era), adjusted for geographic variability, $\Delta(\delta^{13}\bar{C}_{adj})$. All values in per mil (‰). Propagated uncertainties are reported for $\Delta(\delta^{13}\bar{C}_{adj})$ at the 1σ level.

Model	$\Delta(\delta^{13}\bar{C}_{adj})$	$\Delta(\delta^{13}\bar{C}_{MAP})$	$\Delta(\delta^{13}\bar{C}_{pCO_2})$
Plants (all)	–2.77	–0.13	–2.63 ± 1.87
Plants (gymnosperms)	–1.65	–0.27	–1.38 ± 1.21
Plants (North America)	–1.86	–0.46	–1.39 ± 2.10
Fauna (all)	–0.93	–0.40	–0.53 ± 1.52

Supplementary References

[1] Schubert, B. A. & Jahren, A. H. The effect of atmospheric CO_2 concentration on carbon isotope fractionation in c3 land plants. *Geochimica et Cosmochimica Acta* **96**, 29–43 (2012).

[2] Voelker, S. L. *et al.* A dynamic leaf gas-exchange strategy is conserved in woody plants under changing ambient co2: evidence from carbon isotope discrimination in paleo and CO_2 enrichment studies. *Global change biology* **22**, 889–902 (2016).

[3] Farquhar, G. D., O'Leary, M. H. & Berry, J. A. On the relationship between carbon isotope discrimination and the intercellular carbon dioxide concentration in leaves. *Functional Plant Biology* **9**, 121–137 (1982).

[4] Keeling, C. D. *et al.* Exchanges of atmospheric CO_2 and $^{13}CO_2$ with the terrestrial biosphere and oceans from 1978 to 2000. i. Global aspects. *Scripps Institution of Oceanography* (2001).
