# Supplementary material for: Atmospheric CO2 effect on stable carbon isotope composition of terrestrial fossil archives
Source: Nat Commun. 2018 Jan 17;9:252. doi: 10.1038/s41467-017-02691-x (PMC5772509; doi:10.1038/s41467-017-02691-x)
Supplement: Supplementary file 3 — Description of Additional Supplementary Files [file 41467_2017_2691_MOESM3_ESM.pdf]

Description of Additional Supplementary Files

File Name: Supplementary Data 1
Description: Excel spread sheet containing all ice core data, splines and model outputs (155 kyr to present), and radiocarbon-dated plant cellulose and faunal collagen data (40.6 kyr cal BP to present), including MAP outputs from GCMs for each locality.
